# Active Evasion of CTL Mediated Killing and Low Quality Responding CD8+ T Cells Contribute to Persistence of Brucellosis

**DOI:** 10.1371/journal.pone.0034925

**Published:** 2012-04-25

**Authors:** Marina Durward, Girish Radhakrishnan, Jerome Harms, Claire Bareiss, Diogo Magnani, Gary A. Splitter

**Affiliations:** 1 Department of Pathology and Laboratory Medicine, School of Medicine and Public Health, University of Wisconsin-Madison, Madison, Wisconsin, United States of America; 2 Department of Pathobiological Sciences, School of Veterinary Medicine, University of Wisconsin-Madison, Madison, Wisconsin, United States of America; National Council of Sciences (CONICET), Argentina

## Abstract

Brucellosis is a common zoonotic disease that remains endemic in many parts of the world. Dissecting the host immune response during this disease provides insight as to why brucellosis is often difficult to resolve. We used a Brucella epitope specific in vivo killing assay to investigate the ability of CD8+ T cells to kill targets treated with purified pathogenic protein. Importantly, we found the pathogenic protein TcpB to be a novel effector of adaptive immune evasion by inhibiting CD8+ T cell killing of Brucella epitope specific target cells in mice. Further, BALB/c mice show active Brucella melitensis infection beyond one year, many with previously unreported focal infection of the urogenital area. A fraction of CD8+ T cells show a CD8+ Tmem phenotype of LFA-1hi, CD127hi, KLRG-1lo during the course of chronic brucellosis, while the CD8+ T cell pool as a whole had a very weak polyfunctional cytokine response with diminished co-expression of IFN-γ with TNFα and/or IL-2, a hallmark of exhaustion. When investigating the expression of these 3 cytokines individually, we observed significant IFN-γ expression at 90 and 180 days post-infection. TNFα expression did not significantly exceed or fall below background levels at any time. IL-2 expression did not significantly exceeded background, but, interestingly, did fall significantly below that of uninfected mice at 180 days post-infection. Brucella melitensis evades and blunts adaptive immunity during acute infection and our findings provide potential mechanisms for the deficit observed in responding CD8+ T cells during chronic brucellosis.

## Introduction

*Brucella* spp. are the cause of the most common zoonotic disease in man. There are approximately 500,000 new cases diagnosed each year, with endemic disease flourishing in the Middle East, Mediterranean basin, Northern Africa, and across the Asian continent. Human brucellosis is grossly under diagnosed as its symptoms are frequently similar to influenza and malaria, among others (i.e., fever, fatigue, headaches, general malaise, and myalgia) [1]. Vaccine development has continued for decades with limited success [2], [3], [4]. Recent advances in immunological methods and technology have made it possible to dissect the correlates of protective immunity in brucellosis, bringing much needed hope to the collective vaccine effort.

The immune response to *Brucella* infection is extremely varied and depends on the host, species or strain of *Brucella*, and environment. *B. melitensis* is a facultative, intracellular, and Gram-negative bacteria that is not contained by innate immunity [5]. The antibody response can lower the level of initial infection by the production of IgG opsonins but has little effect on the intracellular phase of *Brucella*. Macrophages kill 90–95% of all phagocytosed bacteria, which would appear to be a successful innate response, yet once the surviving few reach their replicative niche, successful infection of the host is all but guaranteed [6]. Over its extremely long history as a human pathogen, *Brucella* has evolved some impressive and redundant mechanisms to evade innate immunity [7], [8]. These include blocking activation of NFκ-B by mimicking a host protein, producing a non-reactive LPS, using phagosome acidification to its advantage, and inhibiting phagosome-lysosome fusion [9], [10], [11], [12], [13], [14]. These known mechanisms along with additional unknown disruptors of the adaptive immune response contribute to its low infectious dose where 20 bacteria ensure an ID_90_ in humans [15]. There remain many unknowns regarding the success or failure of the adaptive cell mediated response during active or chronic brucellosis. Although CD8+ T cells are considered critical to resolution of intracellular bacterial infections, few details are known as to why *Brucella* spp. persist in the presence of CD8+ T cells. Uncovering more of the particulars of the CD8+ T cell mediated response, for example specific surface phenotypes, factors produced, cellular interactions, and cytotoxic T cell killing of cells expressing *Brucella* peptides will provide needed insight for successful and safe vaccine design [16], [17], [18].

**Figure 1:**
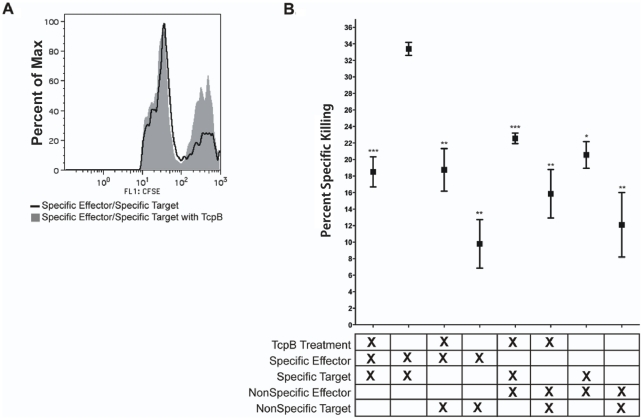
The affect of TcpB on CD8+ T cell specific killing. A) Representative histogram of specific killing after an *in vivo* killing assay with TcpB or maltose binding protein treatment of target cells. Epitope specific peak stained CFSE^hi^ is protected after TcpB treatment of target cells. B) The percent specific killing determined after an overnight incubation of target cells with TcpB or maltose binding protein and then pulsed with *Brucella melitensis* specific epitope. The X in each column on the x-axis indicates the presence of treatment, type of effectors, or target cells in each row. ***p<0.001, **p<0.01, *p<0.05.

By limiting host cell death, an intracellular pathogen can prevent its own exposure to immune surveillance [19]. Infection with live *Brucella* leads to the prolonged life of host macrophages which are protected from apoptosis [5], [20], [21], [22]. Work in our lab on a cell permeable protein of *B. melitensis*, TcpB, has shown its targets to include multiple phosphatidylinositols, particularly those involved at the immunological synapse on the target cell side [13], [14]. When phosphatidylinositol (4,5) bisphosphate (PI(4,5)P_2_) is sequestered away from the synapse, the target cell is rendered resilient against CD8+ T cell mediated killing [23]. The functional capacity of CD8+ T cell effectors and the size of the memory pool can be dependent upon early antigenic, costimulatory, and inflammatory signals [17], [18], [24]. By inhibiting the killing of infected cells in vivo in the presence of specific cytotoxic cells, *B. melitensis* could effectively dampen the immune response, an insult resulting in a smaller and ineffective long-lived memory pool.

There have been recent advances in the broad understanding of CD8+ T cell memory (CD8+ T_mem_), recall responses, and exhaustion [25], [26], [27], [28], [29], [30]. CD8+ T_mem_ cells exhibit stem cell like properties for example longevity, telomerase expression, self-renewal, and a multipotent state that is poised for activation [31]. Also, proliferative capacity of long-lived CD8+ T cells correlates with long-term vaccine efficacy [32], and the recall response mediated by these cells confers protection to a wide variety of infections [33]. A pathogen that evades or disables this response can successfully live long-term within the host, virtually undisturbed. Exploiting the recall response to overcome pathogenic insult via vaccine design may enable researchers to finally protect against low-level reactivating chronic *Brucella* infection.

During acute infection T cells have tight control of cytokine expression, with the ability for rapid on/off regulation [34]. Conversely, in chronic viral infection, T cells are present, but long-lived CD8+ cells lose their ability to produce multiple inflammatory cytokines in a stepwise manner as infection persists [35], [36], [37]. In-depth phenotyping and dissection of the multifunctional Th1 component of non-viral infections is underway, with recent work done in *Mycobacterium*, *Salmonella*, and *Leishmania*, among others [38], [39], [40]. This direction of investigation is crucial in understanding *Brucella* pathogenesis because functional cytokine exhaustion can lead to reactivation of senescent disease with the loss of immune control, as chronic presentation of antigen can induce CD8+ T cell tolerance [24], [37]. In human brucellosis, after intense antibiotic treatment of acute infection, reactivation of disease occurs unpredictably, even in otherwise healthy adults [41], [42], [43]. Brucellosis-acquired cellular anergy has long been noted; however, the mechanism behind this phenomenon is not understood [44]. Here we address the extent of CD8+ T cell memory phenotype and cytokine expression in the host response to chronic *B. melitensis* infection. Our overarching hypothesis is a defect or deficit in the quality of CD8+ T cell response to *Brucella* infection leads to a loss of functional CD8+ T memory cells permitting long-term *Brucella* survival.

Interestingly, a *B. melitensis* derived protein, TcpB, which possesses cell permeable properties, inhibits CD8+ T cell killing of pathogen specific targets. This stunting of the early adaptive response may play a role in the deficit seen in immunological memory during chronic brucellosis. In brucellosis research, BALB/c and C57BL/6 mice are considered prototypal susceptible and resistant animal models, respectively, where the primary site of infection is the spleen [6]. Here we show that the BALB/c mouse model of brucellosis more closely mimics chronic human infection than previously appreciated. We have unexpectedly found that BALB/c mice do not clear the infection and in fact, remain infected for more than 1 year. Surprisingly, *B. melitensis* can be cultured from BALB/c spleens up to 9 months post-infection and the CFUs show an undulation above and below the level of detection over time. The *Brucella* responding CD8+ T cells lack multiple cytokine production (polyfunctionality) while few cells retain a surface memory phenotype in the face of chronic infection. These CD8+T cell findings suggest an exhausted phenotype that would promote long-term *Brucella* survival.

**Figure 2:**
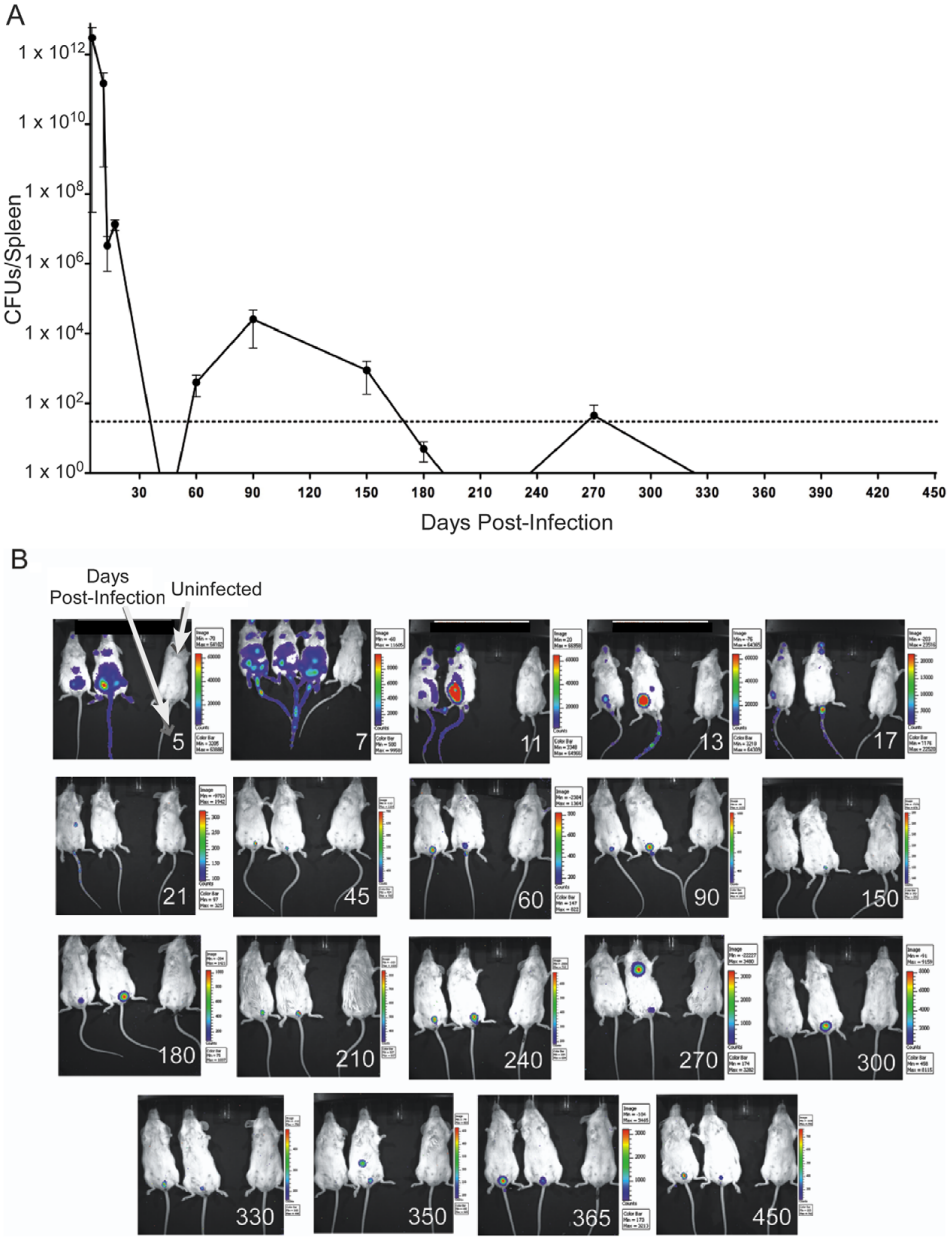
Persistence of infection. BALB/c mice were infected with 10^7^ bioluminescent *Brucella melitensis* GR023**.** A) Colony forming units were determined by culturing serial dilutions of lysed splenocytes for 3–5 days at 37°C. B) Images were captured with a ten minute exposure on an IVIS Imaging System. The rainbow scale of luminescence for each image is in approximate photons. Age-matched control mice are shown in the rightmost position of each individual photo. The day post infection when the images were collected is noted in the bottom right corner of each frame.

## Results

### TcpB is a novel effector of adaptive immune evasion by *Brucella melitensis*

We have previously shown that *B. melitensis* TcpB suppresses inflammatory cytokines of the host including TNF-α and IL-1β [13], [14]. There is recent evidence that targets of TcpB binding, particularly the phosphoinositides, are involved in effector to memory transition signaling in CD8+ T cells as well as involved at the immunological synapse during directed CD8+ T cell killing of target cells [23], [27], [28], [45], [46]. To investigate the involvement of TcpB from *B. melitensis* in CD8+ T cell mediated killing, we performed an in vivo killing assay as described [47]. Briefly, naïve splenocytes were pulsed with the *B. melitensis* peptide NGSSSMATV with TcpB or maltose binding protein as a control and labeled with high Carboxyfluorescein succinimidyl ester (CFSE) 5 µm [16]. Control splenocytes were pulsed with an irrelevant peptide of GFP with our without TcpB and labeled with a low amount of CFSE, 0.5 µm. Equal amounts of CFSE^hi^ and CFSE^lo^ cells were combined and transferred (∼1×10^7^ total cells/mouse) via retroorbital injection to anaesthetized syngeneic mice that had been peptide immunized 7 days prior. After 6 hrs spleens were removed from the mice and examined by flow cytometry for the expression of CFSE and compared to CFSE expression prior to adoptive transfer.

**Figure 3:**
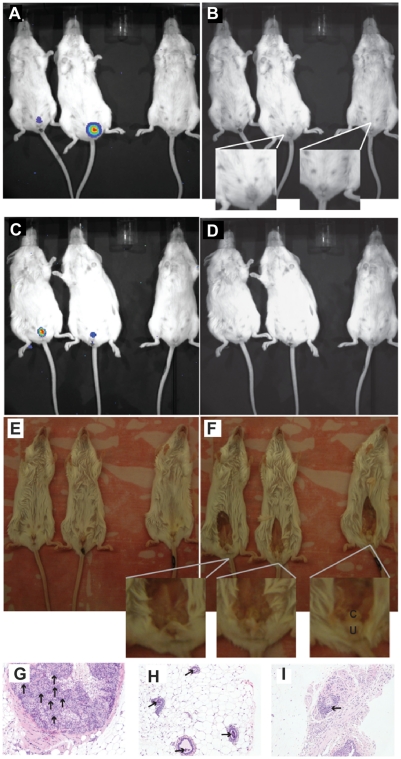
Infection localization. A) Mice infected with bioluminescent *B. melitensis* GR023 shown after luminescence imaging. B) The same image as (A) with the bioluminescence removed to show edema in the urogenital area. The uninfected mouse is on the right side of the frame. A close up of the infected mouse with edema and the control mouse are shown. Mice infected for 450 days are shown with C) bioluminescence, D) with bioluminescence removed, E) just prior to dissection of urogenital area, and F) after dissection showing the clitoral gland (c) just subcutaneous of the ureter (u). Female BALB/c mice infected for 240 days at 20× magnification with infiltration of neutrophils, lymphocytes and macrophages shown by arrows in the G) interstitial region of the clitoral gland, H) lumen of the mammary glands, and I) perivascular cuffing in the subcutaneous tissue of the perianal area.

Interestingly, cells treated with TcpB were significantly protected from specific CD8+ T cell killing (Fig. 1A and B, p<0.001). This observation supports our previous findings that a deletion mutant of TcpB in *B. melitensis* produces a delay in mouse infection as well as elevated TNFα and interleukin-1β in TcpB-deficient *Brucella* compared to wild type bacteria [13]. This suggests that TcpB is a novel effector of adaptive immune evasion by an intracellular bacterial pathogen. By unknown mechanisms, the ability of the pathogen to block responding CD8+ T cell killing of specific targets may contribute to a long-term deficit in immunological memory.

**Figure 4:**
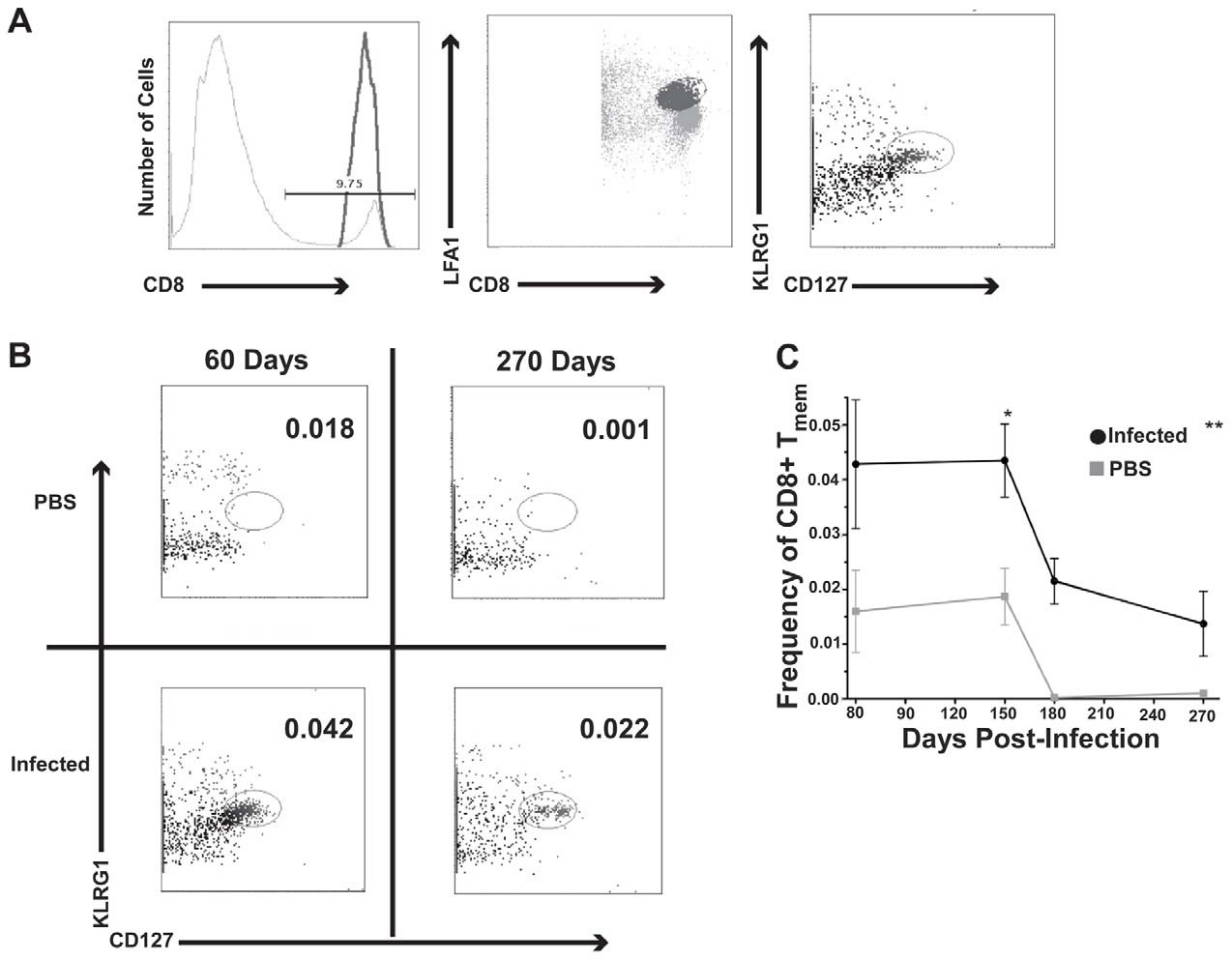
Phenotyping CD8+ T_mem_. A) Representative flow cytometric gating strategy of splenic T_mem_, CD8+ LFA1^hi^CD127^hi^KLRG1^lo^ at day 60 post-infection. From left to right the panels show gating of CD8+ cells, LFA1^hi^CD8+ cells, and CD8+ LFA1^hi^CD127^hi^KLRG1^lo^ cells from analysis of 500,000 to 1 million cells. B) Plots showing the absence of T_mem_ in age-matched uninfected mice (PBS injected) and the presence of T_mem_ in mice with chronic brucellosis. C) Frequency of splenic CD8+ T_mem_ over time. ANOVA was performed to determine if infection with *B. melitensis* had a statistically significant impact on the presence of CD8+ Tmem. **p<0.002 for infected animals at the different days except for day 150 where p<0.05.

### BALB/c mice remain persistently infected with virulent *Brucella melitensis*

Mice (N = 200), in groups of 4, were infected with bioluminescent *B. melitensis* and bacterial load of the spleen was evaluated when mice were sacrificed at specific times post-infection (Fig. 2A). The undulation of bacteria above and below the level of detection over time suggests similarities to human brucellosis. Bioluminescent colonies continued to be cultured from the spleens of infected mice 270 days post-infection demonstrating the chronic nature of infection. No colonies were cultured from the spleens of uninfected age-matched mice kept in separate micro-isolator cages at any time during the experiments (data not shown).

**Figure 5:**
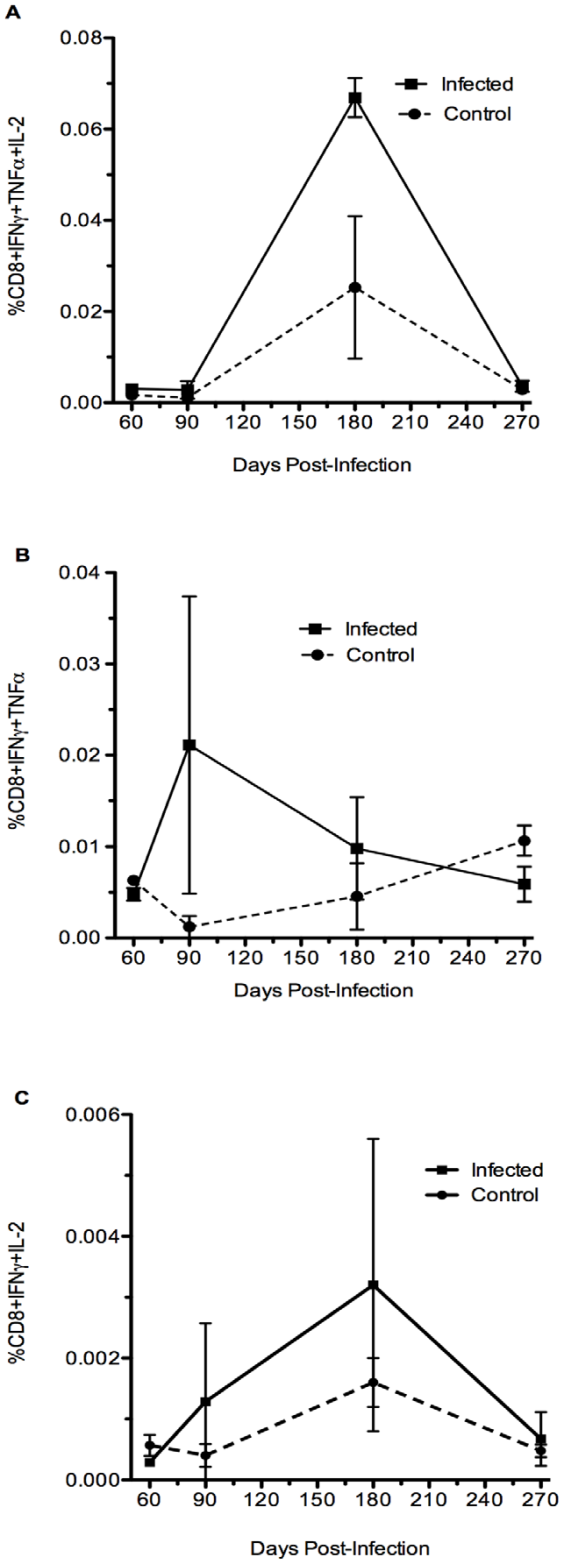
Cytokine co-expression profile of CD8+ T cells expressing 3 or 2 cytokines simultaneously. A) Frequency of polyfunctional splenic CD8+ T cells co-expressing IFN-γ, TNF-α, and IL-2. Expression of 3 cytokines from infected animals was not different from PBS animals over the time of infection, p≤0.06 at 180 days. B) Frequency of dual-function CD8+ T cells co-expressing IFN-γ and TNF-α, p≤0.7 on any day. C) Frequency of dual-function CD8+ T cells co-expressing IFN-γ and IL-2, p≤0.8 on any day.

Infected mice were also monitored for bacterial load prior to death via biophotonic imaging (Fig. 2B). Uninfected age-matched mice are shown on the right of each image. Bacterial luminescence was detected at 450 days post-infection, though becoming barely detectable at times, i.e. 150 days post-infection. Interestingly, biophotonic imaging was more sensitive in detecting infection after 270 days post-infection than the classical method of culturing bacteria from infected mice. Consistent with chronic human reactivating brucellosis, “hotspots” of infection outside of the usual bioluminescent urogenital location were observed in these mice long after acute infections. Note the central chest infection at 270 days post-infection and abdominal infection at 350 days post-infection.

**Figure 6:**
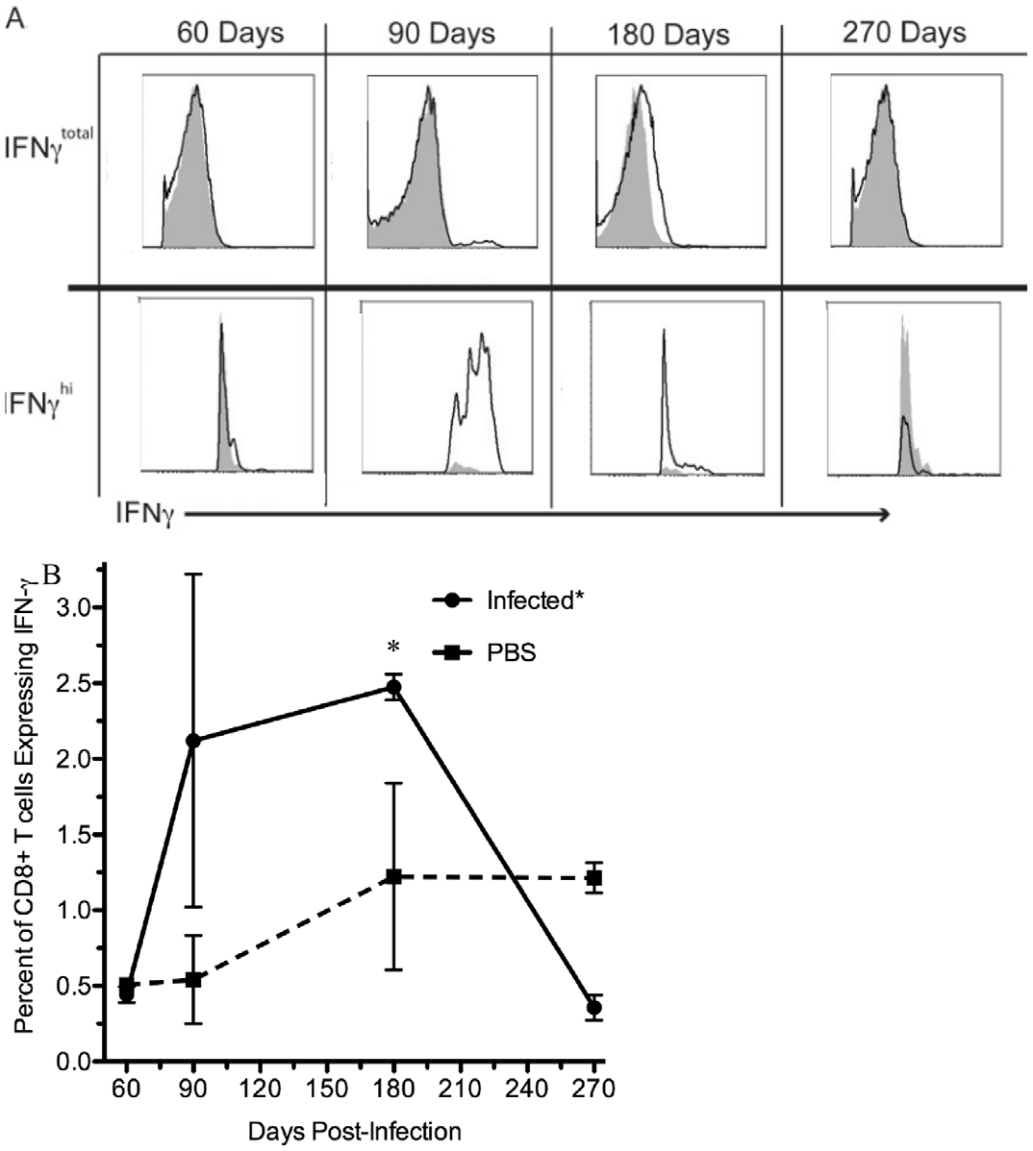
CD8+ T cell IFN-γ expression. A) Histograms of IFN-γ staining over time. Note the top row shows the total IFN-γ expression while the bottom row examines the subtle differences of IFN-γ^hi^ expression between uninfected and infected mice. The black line represents splenocytes from infected mouse pulsed with *B. melitensis* specific peptide. The shaded grey region represents splenocytes from an uninfected age-matched mouse pulsed with *B. melitensis* specific peptide. B) Percent of CD8+ T cells expressing IFN-γ in infected and non-infected mice. *p<0.05.

The urogenital localization of bioluminescence was accompanied in some cases by edema (Fig. 3A–B), redness, and/or alopecia. The murine clitoral gland was consistent with the anatomic luminescence (Fig. 3C, D, E, F). Microscopically, an infiltration of mononuclear cells consistent with macrophages and lymphocytes was observed in the lumen of the gland. Occasional cells with pyknotic nuclei were present in the lining of the gland (Fig. 3G, H, I). Further study is required to determine if the cells in the gland or lumen contain bacteria.

### CD8+ T cells with a memory phenotype are detectable in chronically infected hosts

Because brucellosis remains in a chronic state in BALB/c mice, the presence of a detectable CD8+ T_mem_ cell phenotype in these mice was uncertain. To address this void in our understanding, a combination of antibodies specific for CD3, CD8, LFA1, CD127, and KLRG1 was used to interrogate splenocytes. These markers can be used to distinguish naïve CD8+ T cells (LFA1^lo^ CD127^hi^ KLRG1^lo^), early effectors (LFA1^hi^ CD127^lo^ KLRG1^lo^), short-lived effectors (LFA1^hi^ CD127^lo^ KLRG1^hi^), and CD8+ T_mem_ (LFA1^hi^ CD127^hi^ KLRG1^lo^) [48], [49], [50], [51]. A distinct population of T_mem_ cells was detected in the *B. melitensis* infected mice (Fig. 4A). Analysis of the data revealed that infection with *B. melitensis* does have a very significant impact on the presence of T_mem_ cells, p<0.002 (Fig. 4B and C). Additionally, over time there was a downward trend of T_mem_ cells in infected and age-matched control mice that was significant, p<0.008 (Fig. 4C), most likely reflecting an age-related decline in immune function of older mice. Other controls included cells from infected and uninfected mice pulsed with irrelevant peptide and cells pulsed with no peptide (data not shown). The data were not significantly different from the uninfected age-matched splenocyte data at any time point. In summary, CD8+ T_mem_ are generated and maintained in mice with chronic brucellosis at significantly higher levels than observed in uninfected age-matched control mice.

**Figure 7:**
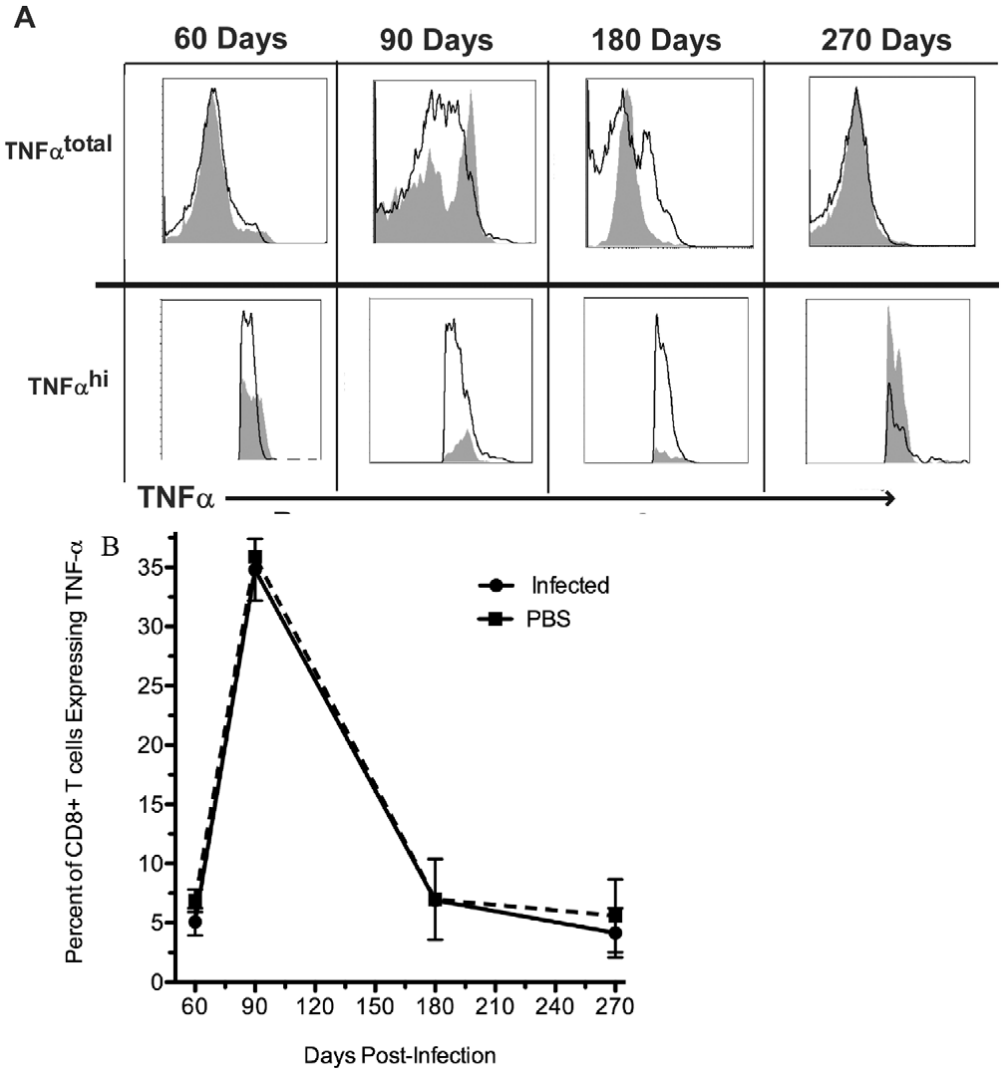
CD8+ T cell TNF-α expression. A) Histograms of TNF-α staining over time. Note the top row shows the total TNF-α expression while the bottom row examines the subtle differences of TNF-α^ hi^ expression between uninfected and infected mice. The black line represents splenocytes from infected mouse pulsed with *B. melitensis* specific peptide. The shaded grey region represents splenocytes from an uninfected age-matched mouse pulsed with *B. melitensis* specific peptide. B) Percent of CD8+ T cells expressing TNF-α in infected and non-infected mice.

**Figure 8:**
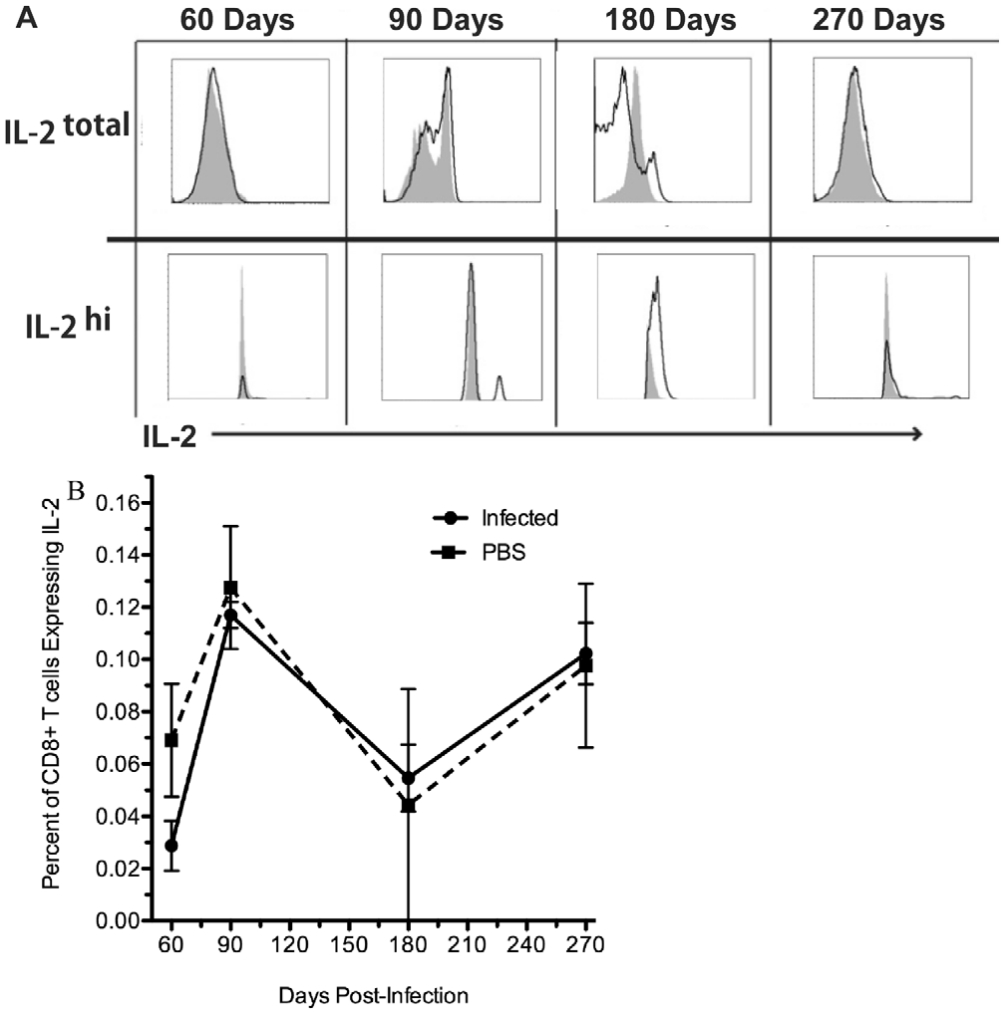
CD8+ T cell IL-2 expression. A) Histograms of IL-2 staining over time. Note the top row shows the total IL-2 expression while the bottom row examines the subtle differences of IL-2 expression between uninfected and infected mice. The black line represents splenocytes from infected mouse pulsed with *B. melitensis* specific peptide. The shaded grey region represents splenocytes from an uninfected age-matched mouse pulsed with *B. melitensis* specific peptide. B) Percent of CD8+ T cells expressing IL-2 in infected and non-infected mice.

### CD8+ T cells are not highly functional during chronic brucellosis

A measure of quality in CD8+ T cells responding to infection, is the ability of a cell to simultaneously produce multiple cytokines [52], [53]. Multifunctional CD8+ T cells have not previously been examined in *Brucella* infection. Here, we examined the ability of *B. melitensis* responding CD8+ T cells to produce IFN-γ in concert with TNF-α and/or IL-2 (Fig. 5). Polyfunctional cells, producing 3 cytokines simultaneously, were near background values at all times examined except for a slight rise at 180 days and are not significant, p<0.06 (Fig. 5A). Dual function cells, either IFN-γ together with TNF-α or IFN-γ together with IL-2, also did not significantly exceed background at any time examined (Fig. 5B and C, p<0.7 and <0.8, respectively) supporting a lack of cytokine function by CD8+ T cells. It remains to be determined whether these cells lose function over time or never establish polyfunctionality during brucellosis.

### IFN-γ expression of CD8+ T cells undulates in chronic brucellosis, while TNF-αα and IL-2 are not expressed significantly above background levels at any time examined

To further investigate the cytokine production of CD8+ T cells during chronic brucellosis, we examined the expression of IFN-γ, TNF-α, and IL-2 independently of one another. Previously, we had shown significant IFN-γ production from CD8+ T cells during acute infection in response to specific *B. melitensis* epitopes [16]. IFN-γ expression of CD8+ T cells from BALB/c mice chronically infected with *B. melitensis* was low at 60 and 180 days post-infection (Fig. 6). Importantly, the cells were unable to respond with IFN-γ production at 270 days post-infection even in the presence of active infection shown by CFUs and biophotonic imaging (Fig. 2), as well as the presence of CD8+ T cells with a memory phenotype (Fig. 4, A, B and C). Infection did have a significant effect (p<0.05) on reducing IFN-γ production when examining CD8+ T cells (Fig. 6). Also, at 60 and 270 days post-infection, not only were the percentages of CD8+ T cells expressing IFN-γ less than PBS treated mice (Fig. 6B), but the geometric mean fluorescence of IFN-γ staining cells in infected mice was significantly less than PBS treated mice (p<0.0002; data not shown), suggesting suppression or lack of IFN-γ production in *Brucella* infected mice. Whether suppression of IFN-γ continues in an undulating fashion beyond 270 days of infection would be of interest in future studies.

**Figure 9:**
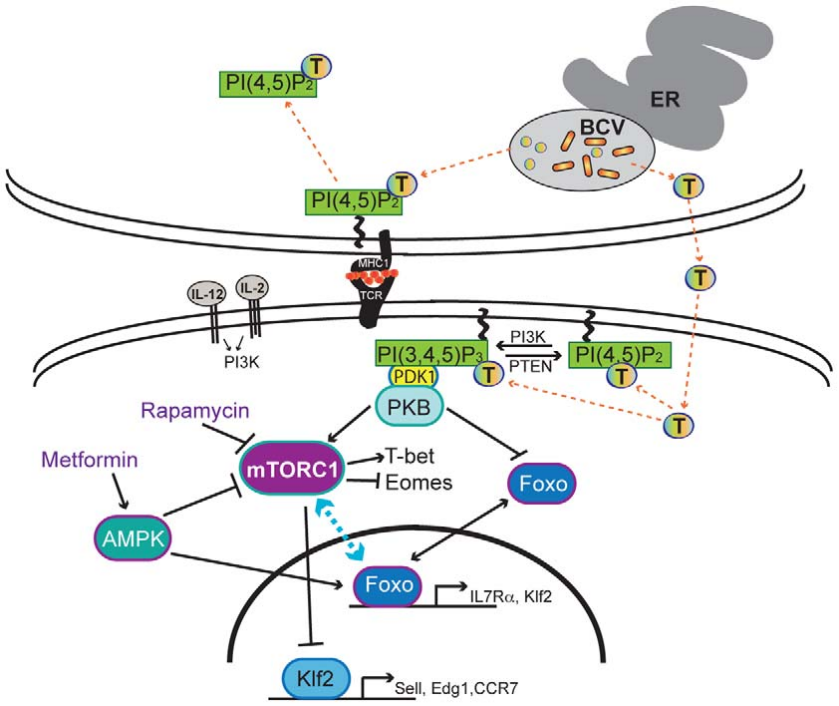
Model of TcpB disruption of adaptive immune response during brucellosis. T =  TcpB; **BCV**  =  *Brucella* containing vacuole; **ER**  =  endoplasmic reticulum. TcpB can inhibit CD8+ T cell specific killing of *B. melitensis* specific target cells. We hypothesize that this inhibition results from the binding of TcpB to PI(4,5)P_2_ and either blocking its action or sequestering it away from the APC side of the immunological synapse. Also, TcpB contains a membrane translocation domain that may allow it to travel into the responding CD8+ T cell. We hypothesize that TcpB binding of PI(4,5)P_2_ and PI(3,4,5)P_3_, TcpB is disrupting by either up-regulating or down-regulating the action of FoxO and/or mTOR. We hypothesize that this disruption leads to decreased numbers of CD8+ T cell making the effector to memory transition necessary for protective immunity. IL-2 and IL-12 activate PI3K which phosphorylates PI(4,5)P_2_ into PI(3,4,5)P_3_. This triggers activation of PKB by PDK1, which can then phosphorylate FoxO and lead to its degradation, and PKB can activate mTOR complex 1. mTORC1 can then up-regulate T-bet, down-regulate Eomes, activate Klf2, and possibly activate FoxO. These actions regulate the fate (short-lived effector or long-lived memory) of the responding CD8+ T cell. Rapamycin and metformin target mTOR and AMPK respectively leading to modulation of memory cell development. Adapted from [17].

TNF-α expression from CD8+ T cells does not significantly fall below or exceed background levels at any time assessed by examining CD8+ T cells as a ratio of splenocytes (Fig. 7A), as a percent of CD8+ T cells (Fig. 7B), and as a mean or geometric mean fluorescence intensity of TNF-α expressing cells (data not shown). Additionally, CD8+ T cell IL-2 expression was never significantly above background during this chronic period of infection (Fig. 8) when examined as a ratio of splenocytes to CD8+ T cells (data not shown), as a percent of CD8+ T cells (Fig. 8B), and as a mean, though IL-2 was significantly lower when examining the mean fluorescence intensity (p<0.05; data not shown). These results suggest that while surface markers showed a memory phenotype, the responding CD8+ T memory cells were of low quality and not functionally resilient. Additionally, *B. melitensis* may not just subvert these immune effectors, but actively suppress the expression of IFN-γ and IL-2 at certain times over the course of infection.

**Figure 10:**
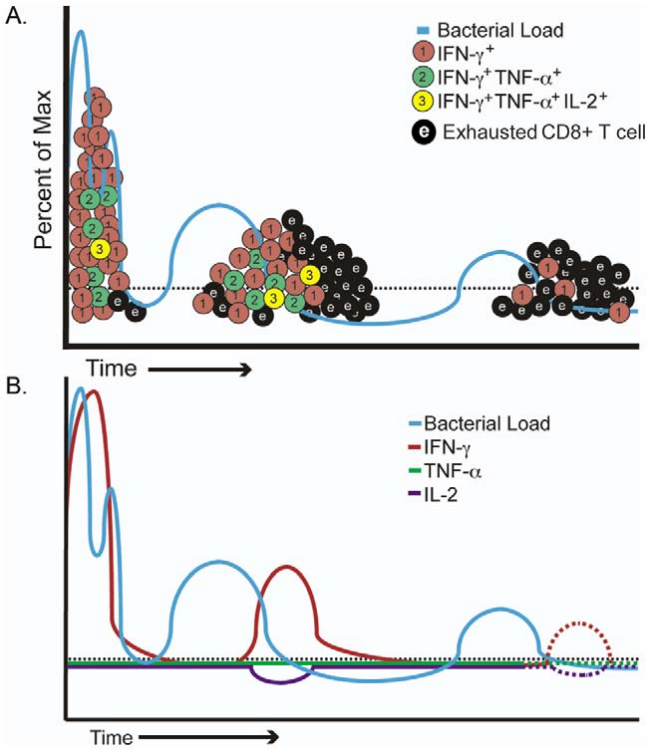
Models of CD8+ T cell function during chronic reactivating brucellosis. A) Model of CD8+ T cell functional response during chronic infection with Brucella melitensis. Initially, CD8+ T cells respond to *B. melitensis* and clonally expand with many clones producing one cytokine such as IFNγ, while fewer clones produce two or three cytokines. With continuing waves of *Brucella* release from infected cells, CD8+ T cell expansion is reduced with fewer and fewer cells producing multiple cytokines and a larger pool of cells producing no detectable cytokines representing an exhausted phenotype. Adapted from [87]. B) Model of CD8+ T cell expression of individual cytokines. Black dotted line represents background level of expression in uninfected mice. As waves of *Brucella* are released from infected cells, cytokine expression occurs with reduction of IFNγ and no IL-2 or TNFα expression.

## Discussion

BALB/c mice have long been used as the susceptible model of brucellosis, yet there remains the uncertainty that *Brucella* infection of mice may not be similar to a natural host [6], [16], [54]. Here, we present evidence of: 1) chronic infection in BALB/c mice for greater than 1 year, 2) a low level of CD8+ T_mem_ cells, 3) lack of polyfunctional cytokine production, and 4) the *B. melitensis* protein TcpB inhibits in vivo cytotoxic CD8+ T killing of *Brucella* peptide expressing target cells. BALB/c mice do not clear *B. melitensis* and show focal sites of reactivation. The undulating nature of the bacterial load should not be surprising considering that human brucellosis has also been known as undulant fever [5]. Histopathological study of the urogenital localization by *B. melitensis* in BALB/c mice compared to its prevalence in humans will provide insight into the local cellular response and environmental cues that allow such long-term persistence in this location [55].

During brucellosis the function of CD8+ T cells may be compromised by bacterial factors that contribute to poor immunological memory. Previous work in our lab had shown that a protein of *B. melitensis*, TcpB, binds to PI(4,5)P_2_ which is required at the immunological synapse on the target cell side for effective CD8+ T cell mediated killing [13], [23]. We observed that TcpB treatment of target cells inhibits *B. melitensis* specific CD8+ T cell killing, evidence that TcpB represents a novel effector of adaptive immune evasion. TcpB has also been shown to dampen NFκ-B activation, working as an effector of innate immune evasion. Also, the *Brucella* NV9 (NGSSSMATV) and RL9 (RYCINSASL) peptides that are recognized by the cytotoxic CD8+ T cell are naturally processed from intact *Brucella* in a mouse infection [16] supporting the relevant role of the CD8+ T cells in the present study.

An important concept that has not been addressed in *Brucella* research, is the effector to memory transition of effector CD8+ T cells. Maximizing the number of effectors that make this transition may improve long-term immunity to *Brucella*. Recently, the role of mammalian target of rapamycin (mTOR) in the effector CD8+ T cell transition to memory has come to light [27], [28]. Also, treatment of LCMV infected mice post-infection with rapamycin, an mTOR inhibitor, surprisingly increased the quantity and quality of LCMV specific CD8+ T cells. This effect was reproduced in vaccinia virus Ankara vaccinated non-human primates [27]. The pathogenic protein TcpB of *Brucella*, can bind to phosphatidylinositol (4,5) bisphosphate (PI(4,5)P_2_) and PI(3,4,5)P_3_ which are present on the APC side of the immunological synapse [13], [23]. Also, both PI(4,5)P_2_ and PI(3,4,5)P_3_ lie upstream of a pathway of mTOR activation in CD8+ T cells [17]. TcpB has a membrane translocation domain and may traffic between infected and uninfected cells [14]. Fig. 9 shows our hypotheses related to the action of TcpB and adaptive immunity. *B. melitensis* produces TcpB that contains a membrane translocation domain that permits its movement from the *Brucella* containing vesicle to the cytosol of the infected cell [14]. TcpB binds to PI(4,5)P_2_ either blocking its action or sequestering PI(4,5)P_2_ away from the APC side of the immunological synapse leading to the failure of CTL lysis of cells expressing *Brucella* peptides in the context of MHCI [23]. Also, the membrane translocation domain of TcpB would allow trafficking of TcpB to responding CD8+ T cells where it could disrupt FoxO and/or mTOR decreasing the transition of effector CD8+ T cells to memory T cells [17]. The resulting decline in functional CD8+ T memory cells is similar to our current findings. The level of TcpB expressed *in vivo*, whether TcpB is actively secreted, or if it becomes free in the cell after bacterial death are unknown. Additionally, determining if TcpB is disrupting, activating, or otherwise manipulating the mTOR activation signaling pathway may elucidate a mechanism behind the lack of protective memory seen in many *Brucella* species/host interactions.

The importance of inducing cellular immunity to intracellular pathogens is well established [56], [57], [58], [59], [60], [61]. A successful vaccine must elicit high quality immunological memory. Murine brucellosis is markedly exacerbated in β2-microglobulin knockout mice that lack CD8+ T cells compared to CD4+ T cell deficient mice or C57BL/6 (wild type) mice, illustrating the predominant role of MHC class I-restricted T cells in controlling *Brucella* infection [62]. Additionally, recent work continues the dissection of the cytokine and CD8+ T cell component of the host response to *Brucella* infection [63]. This is indicative of the importance of cytotoxic CD8+ T cells in a protective immune response against *Brucella* infection. There have been extensive *Brucella* spp. vaccine candidates, but minimal attempts to characterize CD8+ T_mem_ that would be required to engender protection against brucellosis. Determining CD8+ T_mem_ presence and quality during chronic infection was undertaken to enable a needed measure of vaccine efficacy in future studies. Evaluating CD8+ T_mem_ when different *Brucella* strains and species are compared would provide insight into virulence and persistence of such strains and species. Future work will also address the adoptive transfer of memory and challenge experiments to further define the correlates of protective immunity.

IFN-γ is the cytokine best characterized for its involvement in brucellosis [64], [65], [66], [67]. A deficiency of IFN-γ enhances the susceptibility of mice to *Brucella* infection and animals die at ∼10 weeks of infection [65]. Also, DNA immunization with a *Brucella* gene produced high IFN-γ production but no protection and heat-killed *Brucella* sp. induce IFN-γ and IL-2 but not protective cytotoxic T cells [68], [69]. Here, we focus on the role that IFN-γ has as a measure of quality in CD8+ T_mem_. Multifunctional cells, those expressing IFN-γ, TNF-α, and IL-2 simultaneously, are of central importance in the generation of durable protective CD8+ T cell memory. Experiments done with *Mycobacterium tuberculosis* and the *Mycobacterium bovis* BCG vaccine have shown an increase in the quality of the T cell mediated response to challenge when polyfunctional cells are present [70].

In Fig. 10, we introduce a model of CD8+ T cell functionality over the course of chronic reactivating brucellosis. The level of exhausted CD8+ T cells is based on our present cytokine findings that CD8+ T cells express only a single cytokine (IFNγ) and not additionally important cytokines like TNFα or IL-2 similar to exhausted CD8+ T cells. Similar exhaustion of cytokine production by CD8+ T cells is reported for other pathogens [17], [52], [53], [71], [72], [73]. Determining additional cell surface markers as well as transcription factor expression of the CD8+ T memory cells would further define this CD8+ T cell phenotype. Exhausted T cells tend to show a surface phenotype similar to effector T cells, including CD43^hi^, CD69^hi^, CD62L^lo^, CD127^lo^, and inhibitory receptors [71], [72], [73], [74], [75], [76], [77]. Inhibitory receptors PD-1 and LAG-3 have recently become important targets of research, for example blocking the PD-1/PD-1L inhibitory pathway can boost immunity to lymphocytic choriomeningitis virus (LCMV) but conversely, it may be required for a multifunctional protective response to *Salmonella* [35], [40]. This highlights the importance of dissecting the intricacies of pathogen specific cellular responses.

Future brucellosis vaccines will need to maximize high quality T cells that avoid excessive contraction after acute infection and avoid functional exhaustion during the chronic phase in order to induce protective memory. We show that chronic infection of BALB/c with virulent, live *Brucella melitensis* does not establish durable, high quality CD8+ T cell memory. CD8+ T cells responding to long-term infection produce primarily IFN-γ in an undulating pattern that temporally follows the level of bacterial load (Fig. 10). The lack of significant TNF-α expression has ramifications on the function and production of IFN-γ against active infection, which has been documented previously [78], [79]. IL-2 regulates granzyme expression and is important for the expansion of CD8+ T cells; therefore, minimal IL-2 expression would not favor CD8+ T cells responding to reactivation of senescent brucellosis. Endogenous IL-2 is also intimately involved in CD8+ T cell survival and proliferation, and considered a critical aspect of CD8+ T cell mediated protection [80], [81]. This work supports previous studies that have found reduced Th1 immunity in aged mice and humans suffering from brucellosis [82], [83]. Unlike the previous study in aged mice, young mice were infected in the present study and followed for greater than 1 year. Infected young mice have a robust immune system and, in theory, should be able to clear infection. However, such young mice did not clear the bacteria supporting the ongoing chronic nature of brucellosis. Also, during acute murine brucellosis antigen presenting cells could process intact *Brucella* and present the NV9 peptide to CD8+ T cells generating a robust cytotoxic T cell response [16]; however, in the presence of the *Brucella* TcpB protein cytotoxicity is inhibited and as infection progressed cytokine production by CD8+ T cells is reduced.

The findings presented here contribute to understanding the host immunologic response during chronic *B. melitensis* infection. The fact that biophotonic imaging demonstrates BALB/c mice do not clear *B. melitensis* has importance in vaccine and challenge studies. If *Brucella* cannot be cultured from spleens, that does not confirm the mouse is uninfected or has cleared infection. In chronic brucellosis of BALB/c mice the clitoral gland appears to be a site of frequent long-term infection and humans frequently have focal infection of the genital tract [55]. The tropism for the genital organs of ruminants may be related to the high concentrations in these locations of erythritol, a carbohydrate that allows *Brucella* to grow. Although in humans the concentration of erythritol in seminal fluid and prostatic secretions is far lower than that found in ruminants, there are high concentrations of other carbohydrates, which might explain the fact that genitourinary complications are also frequent in humans. Although CD8+ T_mem_ that are CD127+KLRG1^lo^ are present long after initial infection, the cytokine response by these cells is weak suggesting an exhaustive phenotype of CD8+ T_mem_ that may contribute to continuing, chronic *Brucella* infection. Future work will investigate the role of TcpB in evasion of multiple layers of immunity and how this protein affects the lack of strong memory response.

## Materials and Methods

### Brucella melitensis

Bioluminescent *B. melitensis* GR023 [84] was grown in brucella broth (Difco) at 37°C with shakig.

### Immunization of Mice

Female BALB/c mice (6–8 weeks of age) were obtained from Harlan (Indianapolis, IN) and housed in AAALAC approved facilities under pathogen-free conditions using protocols approved by the university animal care committee (number V0554). For *in vivo* killing studies, groups of 4 mice were immunized s.c. at the base of the tail with 50 μg of each peptide in PBS/10% DMSO emulsified 1∶1 in IFA, or IFA alone. For the *Brucella* specific immunological memory studies, groups of 4 mice were immunized intraperitoneal (i.p.) with 10^7^ *B. melitensis* GR023 [84] to ensure establishing long-term infection without causing death. Uninfected age-matched mice were injected i.p. with phosphate buffered saline (PBS) in the place of bacteria. This study was carried out in strict accordance with the recommendations in the Guide for the Care and Use of Laboratory Animals of the National Institutes of Health. The protocol was approved by the Institutional Animal Care and Use Committee of the University of Wisconsin-Madison that evaluates the ethics of animal experiments (Permit Number: V00554). All in vivo imaging was performed under isofluorane gas anesthesia. All efforts were made to minimize animal suffering, and animals were housed in AAALAC accredited facilities.

### In vivo imaging

Mice were evaluated using biophotonic imaging as previously described [84], [85]. Briefly, mice were anaesthetized per AAALAC and institutional guidelines for a ten minute exposure in the IVIS Imaging System (Caliper LifeSciences, Hopkinton, MA). Images were collected and analyzed using Living Image (Caliper LifeSciences, Hopkinton, MA).

### Surface staining and flow cytometry

Splenocytes from immunized and control mice were isolated and immediately stained with anti-CD8 (PerCP-Cy, Beckman Coulter, Fullerton, CA), anti-CD3, anti-LFA1 (a.k.a. CD11a), anti-CD127 (17A2, 2D7, and SB/199, BD Biosciences, San Jose, CA), and anti-KLRG1 (2F1, Abcam, Cambridge, MA). Flow cytometry was performed on an FC500 (Beckman Coulter, Fullerton, CA) with 500,000 to 10^6^ events acquired. Data was further analyzed using FlowJo (Tree Star, Ashland, OR) and Prism (GraphPad Software, La Jolla, CA).

### Intracellular cytokine assay

Splenocytes from immunized and control mice were cultured in 96-well round bottom plates (1×10^6^ cells/well) in complete medium in the presence of 100 μg/ml–0.1 ng/ml of purified MHC Class I peptide (>90% purity, GenScript, Piscataway, NJ), 10 μg/ml GolgiPlug (BD Biosciences, San Jose, CA), with or without Concanavalin A (Sigma-Aldrich, St. Louis, MO). After 5 hrs, cells were surface stained with anti-CD8 and anti-CD3. Cells were treated with Lysis Buffer (BD Biosciences, San Jose, CA) then fixed and permeabilized according to the Cytofix/Cytoperm manufacturers protocol, with a subsequent intracellular stain with anti-IFNγ, anti-TNFα, and anti-IL-2 (BD Biosciences, San Jose, CA). Flow cytometry was performed on an FC500 (Beckman Coulter, Fullerton, CA). Data was further analyzed using FlowJo (Tree Star, Ashland, OR) and Prism (GraphPad Software, La Jolla, CA).

### Protein purification

Expression and purification of TcpB was performed as previously described [14]. Briefly, Overnight grown *E. coli* BL21 cells harboring the pMALTcpB plasmid construct or pMAL vector was inoculated (0.1%) into 1L of LB medium with glucose (2 g) and ampicillin (100 µg/mL). The culture was grown at 37°C and induced with IPTG to a final concentration of 0.5 mM when the OD_600_ reached 0.6. The induced culture was then grown at 25°C for 5 hrs. Cells were collected by centrifugation and resuspended in sonication buffer containing 50 mM Tris-HCI [pH 8.0], 1 M NaCI, 1 mM EDTA and 1X protease inhibitor cocktail (Pierce). Cells were sonicated and then centrifuged at 16000×g for 20 min to clarify the supernatant. The supernatant was passed through a column harboring 5 mL of amylose resin (NEB). The column was then washed with the sonication buffer followed by the same buffer containing decreasing concentrations of NaCI (750, 500, 250 and 100 mM). The bound MBP-TcpB protein was eluted with an elution buffer containing 50 mM Tris-HCI [pH 8.0] and 30 mM maltose. The eluted protein was then subjected to Genenase I protease site to cleave TcpB from MBP followed by SP Sepharose (Sigma) ion exchange chromatography to remove maltose followed by concentration using a centricon protein concentrator (Millipore). The concentrated protein was dialyzed in a buffer containing 50 mM Tris-HCI, 100 mM NaCI and 10 % glycerol and stored at −80°C as aliquots. MBP was used as a negative control.

### In vivo killing assay

Splenocytes from naive BALB/c mice were cultured overnight with 50 µg/ml of purified TcpB or MBP as a negative control. As described previously [16], [47], control and experimental splenocytes were then labeled with 5.0 μm or 0.5 μm CFSE (high and low concentrations, respectively). CFSE^lo^ cells were pulsed with irrelevant peptide, GYKVAPAAL, (1 μg/ml) and CFSE^hi^ cells were pulsed with NGSSSMATV (1 μg/ml) for 2 hrs prior to CFSE staining. Equal amounts of CFSE^hi^ and CFSE^lo^ cells were combined and transferred (∼1×10^7^ total cells/mouse) via retroorbital injection to anaesthetized syngeneic mice that had been peptide immunized 7 days prior. After 6 hrs, splenocytes were analyzed by flow cytometry for the presence of hi and lo CFSE-labeled cells. The percent killing was calculated as (1-(Ratio of Irrelevant:Epitope specific cells in naïve mouse/Ratio in immunized mouse)) ×100 [86].

### Statistical analysis

To determine statistical significance, paired Student's t-test and ANOVA were performed on the data against the control using Prism (GraphPad Software, La Jolla, CA). A p value of <0.05 was considered significant.
